# IL-1α and IL-36 Family Cytokines Can Undergo Processing and Activation by Diverse Allergen-Associated Proteases

**DOI:** 10.3389/fimmu.2022.879029

**Published:** 2022-06-30

**Authors:** Valentina Frezza, Zaneta Najda, Pavel Davidovich, Graeme P. Sullivan, Seamus J. Martin

**Affiliations:** Molecular Cell Biology Laboratory, Department of Genetics, The Smurfit Institute, Trinity College, Dublin, Ireland

**Keywords:** IL-1 family, inflammation, allergens, proteolysis, IL-36 cytokine family, IL-1α

## Abstract

Inflammation driven by environmental allergens is an important source of morbidity in diseases such as asthma and eczema. How common allergens promote inflammation is still poorly understood, but previous studies have implicated the protease activity associated with many allergens as an important component of the pro-inflammatory properties of these agents. The IL-1 family cytokine, IL-33, has recently been shown to undergo processing and activation by proteases associated with multiple common allergens. However, it remains unclear whether the sensing of exogenous protease activity—as a proxy for the detection of invasive microbes, allergens and parasitic worms—is a general property of IL-1 family cytokines. In common with the majority of IL-1 family members, cytokines within the IL-36 sub-family (IL-36α, IL-36β and IL-36γ) are expressed as inactive precursors that require proteolysis within their N-termini for activation. Here we show that proteases associated with multiple common allergens of plant, insect, fungal and bacterial origin (including: *Aspergillus fumigatus*, ragweed, rye, house dust mite, cockroach and *Bacillus licheniformis*) are capable of processing and activating IL-36 family cytokines, with IL-36β being particularly susceptible to activation by multiple allergens. Furthermore, extracts from several allergens also processed and enhanced IL-1α activity. This suggests that multiple IL-1 family cytokines may serve as sentinels for exogenous proteases, coupling detection of such activity to unleashing the pro-inflammatory activity of these cytokines. Taken together with previous data on the diversity of proteases capable of activating IL-1 family cytokines, this suggests that members of this cytokine family may function as ‘activity recognition receptors’ for aberrant protease activity associated with infection, tissue injury or programmed necrosis.

## Introduction

Inflammation is typically initiated *via* ‘pattern recognition receptors’ (PRRs) that detect conserved pathogen structures, termed PAMPs (pathogen-associated molecular patterns), that bind to a diverse array of PRR receptors such as TLRs, NLRs, and CTLRs, expressed on sentinel immune cells (1). However, inflammation can also be initiated through stimuli—such as toxins, xenobiotics, blunt force trauma and burns—that severely perturb tissue homeostasis and provoke necrotic cell death (2). The latter stimuli typically result in the liberation of DAMPs (damage-associated molecular patterns), such as members of the extended IL-1 cytokine family, which are potent instigators of inflammation in numerous contexts (reviewed in Martin (3)). However, allergens and helminth worms also elicit robust inflammatory responses, but how these agents are sensed by the immune system to promote inflammation is still poorly understood (4–6).

Emerging evidence suggests that the proteolytic activities associated with common environmental allergens, as well as helminth worms, play a key role in triggering inflammation (7, 8), either by directly acting on Basophils or Mast cells to promote IL-4 release (5, 6), or through proteolytic processing and activating the Th2-polarizing IL-1 family cytokine, IL-33 (9, 10). Thus, in addition to the detection of PAMPs and DAMPs, emerging evidence suggests that inflammation may also be initiated *via* molecules that are capable of sensing aberrant enzymatic activities—such as protease activity—in tissue or cellular compartments where these activities are normally not present (reviewed in Martin et al. (11). Such ‘activity recognition receptors’ (ARRs) may complement PRRs as instigators of inflammation through detecting molecular *activities*, rather than molecular *patterns*, associated with non-self entities (11).

Pathogens and other non-self entities that infiltrate barrier surfaces harbor proteases, as well as other enzymes, that may serve as potent instigators of inflammation through activating ARRs, either directly, or through processing ARR ligands, such as members of the extended IL-1 family (11). IL-1 family cytokines are pleiotropic cytokines released during infection or injury and play key roles as initiators of inflammation (3, 12–14). Members of the IL-1 cytokine family are frequently among the most apical cytokines released during infection or tissue damage and most likely serve as damage-associated molecular patterns (DAMPs) as a result of their release during necrosis (3, 14, 15).

In common with the majority of IL-1 family members, IL-36 cytokines are expressed as completely inactive precursors and require proteolytic processing within their N-terminal pro-domain regions for activation (16–18). The proteases that process and activate these cytokines have only recently been identified and include the neutrophil proteases cathepsin G, elastase and proteinase-3, as well as cathepsin S (17–20). IL-36α, IL-36β and IL-36γ are all encoded by distinct genes and signal through a common receptor, IL-36R, leading to NFκB activation and the production of a broad array of pro-inflammatory cytokines and chemokines from diverse cell types and tissues (16–18, 21–23). IL-36α, IL-36β and IL-36γ are highly expressed in skin and barrier tissues and accumulating evidence has implicated IL-36 cytokines as key mediators in a variety of inflammatory diseases, particularly psoriasis (24–27).

As noted earlier, allergen-associated proteases have been implicated as drivers of the type 2 inflammatory reactions that are typically provoked by such agents (5, 6, 9, 10). It has been shown that IL-33 can act as a sensor for multiple allergen-associated proteases, both *in vitro* as well as *in vivo* (9, 28). A recent report has also shown that IL-36γ can be processed and activated by a protease associated with *Aspergillus fumigatus* (29). However, it remains unknown whether susceptibility to processing by diverse allergen-associated proteases is restricted to IL-33 or is a property shared by multiple IL-1 family members. Here, we have explored the susceptibility of four members of the extended IL-1 cytokine family, including human IL-1α, IL-36α, IL-36β and IL-36γ, to serve as substrates for allergen-associated proteases *in vitro*. We show that human IL-36 family cytokines are differentially processed and activated by proteases associated with multiple allergens of fungal, plant, insect and bacterial origin. IL-36β was especially susceptible to processing and activation by proteases associated with house dust mite (HDM), Cockroach, *Aspergillus fumigatus* and *Bacillus licheniformis*, as well as other common allergens. Mapping of the protease cleavage sites within IL-36β identified Arg5 as the primary site of processing and activation by HDM and Cockroach-associated proteases and mutation of this site completely suppressed IL-36β activation by the latter allergens. Similarly, human IL-1α was also processed and activated by extracts from multiple allergens *in vitro*. Thus, in common with IL-33, IL-1α and IL-36 cytokines may act as proteolytic sensors for the detection of common allergen-associated proteases, coupling the detection of aberrant protease activity to the initiation of inflammation.

## Results

### IL-36 Family Cytokines Are Inactive as Full-Length Precursor Proteins

**Figure 1:**
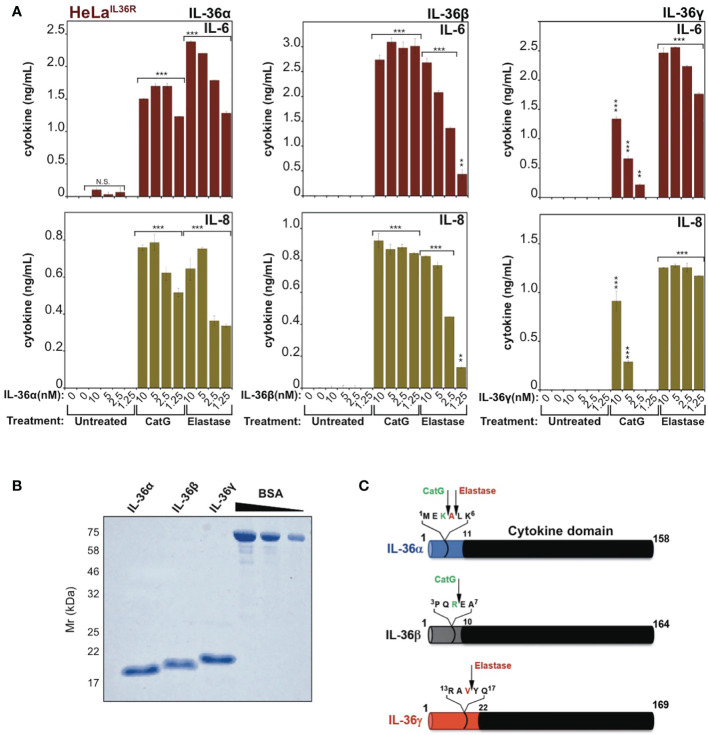
IL-36 family cytokines require proteolytic processing for activation **(A)** HeLa^IL-36R^ cells were stimulated with the indicated dose ranges of recombinant full-length human IL-36α, IL-36β or IL-36γ, that were pre-incubated for 2h at 37°C in the presence or absence of cathepsin G (50nM) or elastase (50nM), as indicated. After 24 h, IL-6 and IL-8 cytokine concentrations in cell culture supernatants were measured by ELISA. **(B)** Human recombinant full-length IL-36α, IL-36β and IL-36γ were purified as described in materials and methods and purity was assesed by SDS-PAGE followed by Coomassie blue staining. **(C)** Schematic representation of human IL-36 family cytokines with cleavage sites for elastase and cathepsin G indicated. Error bars represent the mean of triplicate determinations from representative experiments. ***p < 0.001, **p < 0.01, Student’s t test. N.S., Not Significant.

Previous studies have shown that IL-36α, IL-36β and IL-36γ are completely inactive as full-length proteins (16, 17), but can be processed to their active forms by proteases released from activated neutrophils, such as cathepsin G, elastase and proteinase-3 (17, 18). As illustrated in **Figure 1A**, whereas full-length recombinant IL-36α, IL-36β and IL-36γ failed to elicit inflammatory cytokine production when incubated with an IL-36-responsive cell line (HeLa^IL-36R^), pre-incubation of IL-36 cytokines with either cathepsin G (Cat G) or elastase induced differential activation of IL-36α, IL-36β and IL-36γ, as previously reported (17, 18). Thus, IL-36 family cytokines are inactive as full-length proteins (**Figure 1B**) but become activated upon processing at specific sites within their N-termini (**Figure 1C**).

### Allergen-Associated Proteases Activate IL-36 Cytokines

**Figure 2:**
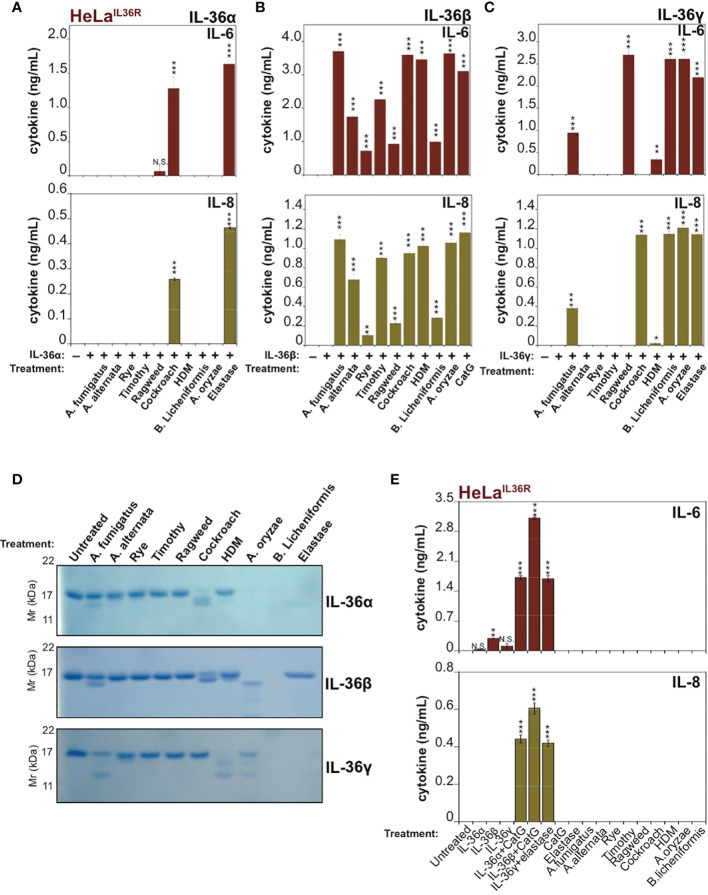
IL-36 family cytokines undergo processing and activation by diverse allergen-associated proteases **(A-C)** HeLa^IL-36R^ cells were stimulated with 2 nM of either full-length human IL-36α, IL-36β or IL-36γ that were pre-incubated for 2 h at 37°C with the indicated allergen extracts (10 μg/mL) or 20 nM of elastase (IL-36α and IL-36γ) or 20 nM CatG (IL-36-β). After 18 h, IL-6 and IL-8 cytokine concentrations in cell culture supernatants were measured by ELISA. **(D)** Recombinant purified human IL-36 family cytokines (4 µg per reaction) were incubated with the indicated allergen extracts (20 μg/mL) or 1:500 stock dilution of purified protease from *A. oryzae*. As a control, IL-36α, IL-36β or IL-36γ were incubated with 50 nM of elastase (IL-36α and IL-36γ) or 20 nM CatG (IL-36β). Reaction products were analized by SDS-PAGE followed by Coomassie blue stain. **(E)** HeLa^IL-36R^ cells were incubated, either alone, or with the indicated allergen extracts (all at 10 μg/mL final concentration, with the exception of *A. oryzae* purified protease which was used at 1:1000 stock dilution). As positive controls, cells were also incubated with 2 nM of either full-length human IL-36α, IL-36β or IL-36γ that were pre-incubated for 2 h at 37 °C with 20 nM of elastase (IL-36α and IL-36γ) or 20 nM CatG (IL-36-β). After 24 h, IL-6 and IL-8 cytokine concentrations in cell culture supernatants were measured by ELISA. Error bars represent the mean of triplicate determinations from representative experiments. ***p < 0.001, **p < 0.01, *p < 0.1, Student’s t test. N.S., Not Significant.

Because recent studies have demonstrated that allergen-associated proteases can promote inflammation in the lung through processing and activation of the IL-1 family cytokine IL-33 (9, 10), we wondered whether other members of the extended IL-1 family were also responsive to allergen-associated proteses. Thus, we explored whether extracts derived from a panel of common allergens could process and activate human IL-36α, IL-36β or IL-36γ. As **Figure 2A** illustrates, incubation of human recombinant full-length IL-36α with the panel of allergen extracts revealed only minor activation of this cytokine by one of the allergens (Cockroach) with none of the other allergens possessing IL-36α-activating activity in this assay. However, although Cockroach extract was the only allergen capable of eliciting significant IL-36α bioactivity, the latter cytokine did undergo proteolysis upon incubation with extracts of *Aspergillus fumigatus*, *Aspergillus orzyae*, and *Bacillus licheniformis* but was not activated under these conditions (**Figure 2A**).

In sharp contrast to the results observed with IL-36α, human recombinant IL-36β was robustly processed and activated by extracts from multiple common allergens, including: *Aspergillus fumigatus, Aspergillus alternata*, Rye, Timothy, Ragweed, Cockroach, house dust mite, *Aspergillus orzyae* and *Bacillus Licheniformis* (**Figure 2B**). Under the same conditions, human IL-36γ was somewhat less susceptible than IL-36β to activation by the majority of allergen extracts, but was robustly activated by extracts from Cockroach, *A. orzyae*, and *Bacillus licheniformis* (**Figure 2C**). SDS-PAGE analysis of IL-36 family cytokines incubated for 2h at 37°C in the presence of the panel of allergen extracts revealed patterns of proteolysis that broadly correlated with the outcome of the bioactivity assays (**Figure 2D**), although it should be noted that trace amounts of proteolysis can often be sufficient to produce highly significant amounts of active cytokine. Interestingly, some of the allergen extracts (such as *A. oryzae* and *B. licheniformis*) appeared to differentially degrade specific IL-36 cytokines to inactivity, while activating others.

Importantly, incubation of HeLa^IL-36R^ cells in the presence of allergen extracts alone failed to induce inflammatory cytokine production, demonstrating that these extracts did not act directly upon HeLa^IL-36R^ cells, or indeed any other medium constituent, to promote inflammatory cytokine production (**Figure 2E**).

**Figure 3:**
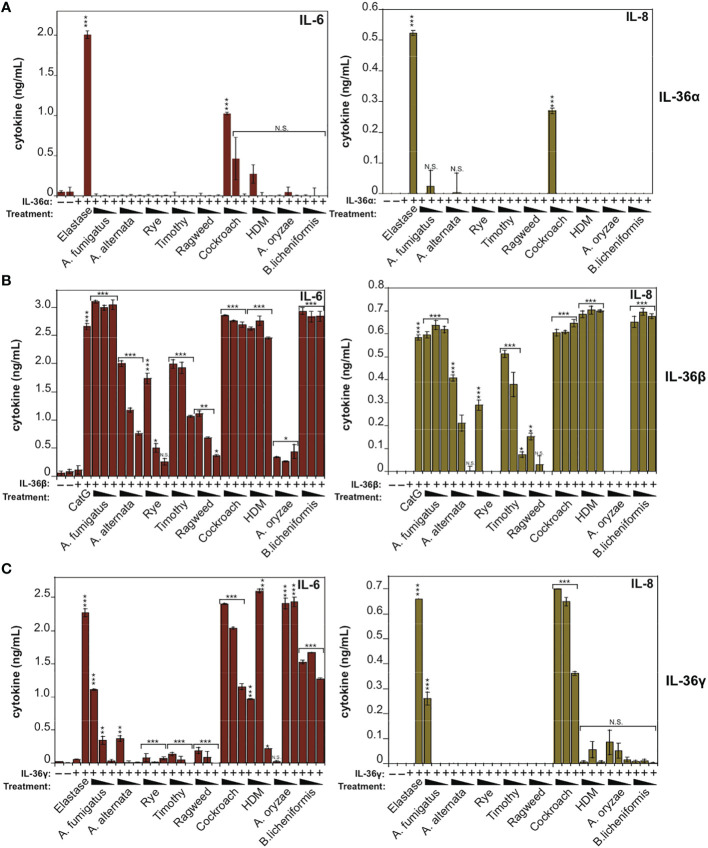
IL-36β is particularly susceptible to activation in response to multiple allergen-associated proteases **(A)** HeLa^IL-36R^ cells were stimulated with full-length human IL-36α (2 nM), either untreated, or after incubation for 2 h at 37°C with the indicated allergen extracts. All allergens were titrated over a concentration range of 20, 10 and 5 μg/ml, with the exception of purified protease from *A. oryzae*, which was used at dilutions of 1:250, 1:500 or 1:1000 of concentrated stock. As a positive control, IL-36α was activated with elastase (20 nM). After 24 h, IL-6 and IL-8 cytokines in cell culture supernatants were measured by ELISA. **(B)** HeLa^IL-36R^ cells were stimulated with full-length human IL-36β (2 nM), either untreated, or after incubation for 2 h at 37 °C with the indicated allergen extracts, as described above. As a positive control, IL-36β was activated with cathepsin G (20 nM). After 24 h, IL-6 and IL-8 cytokines in cell culture supernatants were measured by ELISA. **(C)** HeLa^IL-36R^ cells were stimulated with full-length human IL-36γ (2 nM), either untreated, or after incubation for 2 h at 37°C with the indicated allergen extracts, as described in **(A)**. As a positive control, IL-36γ was activated with elastase (20 nM). After 24 h, IL-6 and IL-8 cytokines in cell culture supernatants were measured by ELISA. Error bars represent the mean of triplicate determinations from representative experiments. ***p < 0.001, **p < 0.01, *p < 0.1, Student’s t test. N.S., Not Significant.

To explore these observations further, we co-incubated IL-36 cytokines with the panel of allergen extracts over a wide range of extract dilutions and, once again, found that hIL-36β was highly susceptible to activation by the majority of allergens examined, in particular by extracts from *A. fumigatus*, cockroach, HDM and *B. Licheniformis* (**Figure 3B**). In agreement with the previous observations (**Figure 2**), hIL-36α was only modestly activated by Cockroach extract (**Figure 3A**) and hIL-36γ was susceptible to activation by a more restricted group of allergens than hIL-36β (**Figure 3C**). Thus, of the three IL-36 family cytokines, IL-36β was highly sensitive to processing and activation by multiple allergen-associated proteases.

### IL-1α Is Also Activated by Multiple Allergen-Associated Proteases

**Figure 4:**
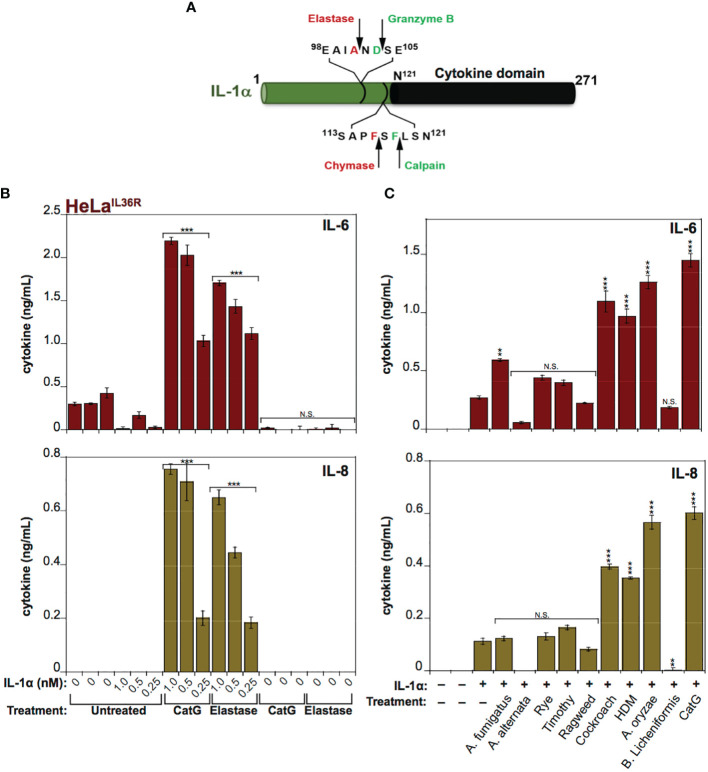
IL-1α activity is enhanced by exposure to neutrophil-derived proteases as well as several allergen-associated proteases **(A)** Schematic representation of human IL-1α with known cleavage sites for elastase, granzyme B, chymase and calpain indicated. **(B)** HeLa^IL-36R^ cells were stimulated with the indicated concentrations of full-length human IL-1α, either untreated, or after incubation for 2 h at 37°C with elastase (25 nM) or cathepsin G (25 nM), as shown. After 24h, IL-6 and IL-8 cytokine concentrations in cell culture supernatants were measured by ELISA. **(C)** HeLa^IL-36R^ cells were stimulated with full-length human IL-1α (2 nM), either untreated, or after incubation for 2 h at 37°C with the indicated allergen extracts (10 μg/ml of all allergen extracts was used, with the exception of *A. oryzae* which was a 1:1000 dilution of stock protease). As a positive control, IL-1α was activated with 20 nM Cathepsin G. After 24 h, IL-6 and IL-8 cytokine concentrations in cell culture supernatants were measured by ELISA. Error bars represent the mean of triplicate determinations from representative experiments. ***p < 0.001, **p < 0.01, Student’s t test. N.S., Not Significant.

IL-1α exhibits a basal level of constitutive biological activity as a full-length cytokine, but this can be enhanced ~10-fold through processing within its N-terminal pro-domain by elastase, cathepsin G, calpain or granzyme B (18, 30, 31) (**Figure 4A**). In agreement with these previous studies, whereas low concentrations (<1 nM) of human recombinant IL-1α exhibited little cytokine activity on HeLa cells, pre-incubation of full-length IL-1α with either Cat G or elastase resulted in a robust increase in IL-1α bioactivity (**Figure 4B**). Furthermore, incubation of human IL-1α with the panel of allergen extracts also resulted in activation of IL-1α by extracts from *A. fumigatus*, cockroach, HDM and *A. oryzae* (**Figure 4C**). In contrast, certain allergen extracts (*A. alternata* and *B. licheniformis*) appeared to inactivate IL-1α under the same conditions (**Figure 4C**).

### Proteolysis of IL-36β and IL-1α by Allergen-Associated Proteases

**Figure 5:**
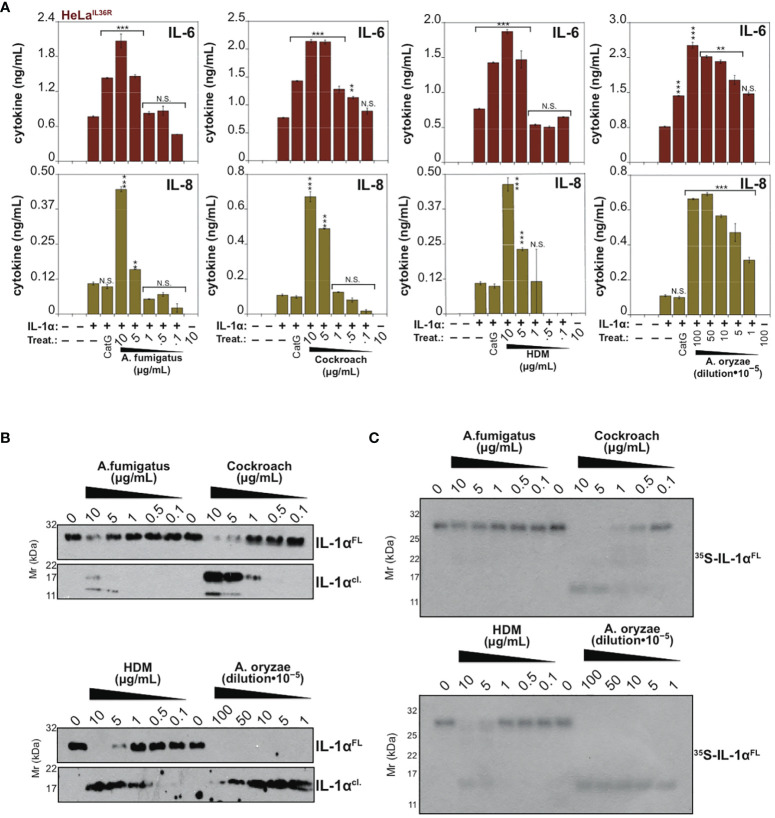
IL-1α is processed and activated by proteases associated with *Aspergillus fumigatus*, cockroach, house dust mite and *Aspergillus oryzae* **(A)** HeLa^IL-36R^ cells were stimulated with full-length human IL-1α (2 nM), either untreated, or after incubation for 2 h at 37°C with the indicated concentrations of allergen extracts. As a positive control, IL-1α, was activated with 50 nM Cathepsin G. After 24h, IL-6 and IL-8 cytokine concentrations in cell culture supernatants were measured by ELISA. **(B)** Recombinant full-length human IL-1α (200 nM per reaction) was incubated for 2 h at 37°C in the presence or absence of a range of concentrations of the indicated allergens, followed by assessment of proteolysis by immunoblotting. **(C)** ^35^S-labeled *in vitro* transcribed/translated human IL-1α was incubated for 2 h at 37°C either alone or with the indicated allergen extracts, followed by SDS-PAGE and fluorography. Error bars represent the mean of triplicate determinations from representative experiments. ***p < 0.001, **p < 0.01, *p < 0.1, Student’s t test. N.S., Not Significant.

**Figure 6:**
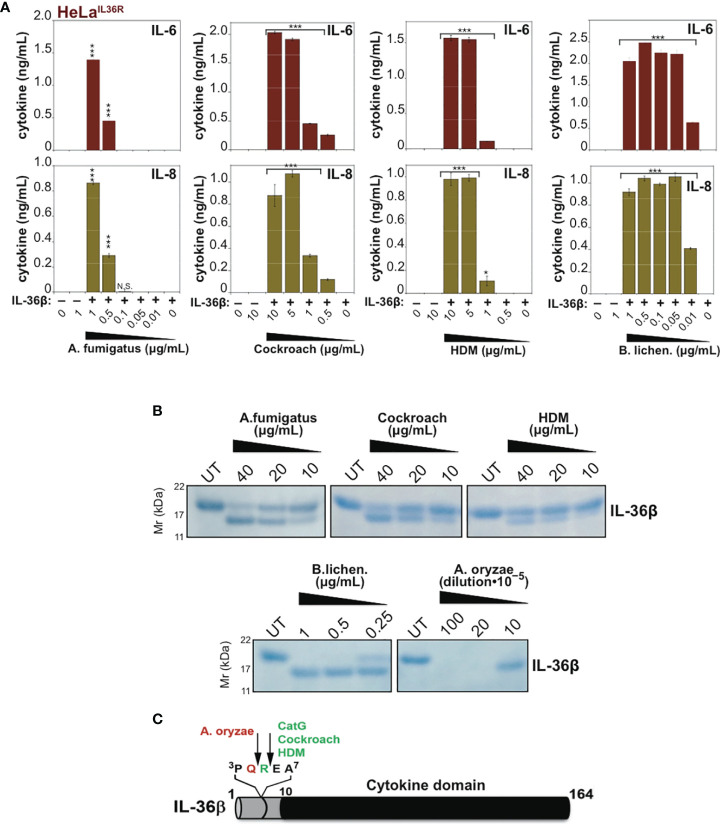
Mapping cleavage sites within IL-36β for proteases associated with *Aspergillus fumigatus*, cockroach, house dust mite and *Bacillus licheniformis.* **(A)** HeLa^IL-36R^ cells were stimulated with 2 nM of full-length human IL-36β, either untreated, or after incubation for 2 h at 37°C with the indicated concentrations of allergen extracts. After 24 h, IL-6 and IL-8 cytokine concentrations in cell culture supernatants were measured by ELISA. **(B)** Recombinant human IL-36β^FL^ (4 µg) was incubated for 2 h at 37°C in the presence or absence of the indicated concentrations of allergen extracts, followed by SDS-PAGE analysis. **(C)** Schematic representation of full-length IL-36β with cleavage sites for cathepsin G and allergen extracts indicated. Error bars represent the mean of triplicate determinations from representative experiments. ***p < 0.001, *p < 0.1, Student’s t test. N.S., Not Significant.

Because IL-36β and IL-1α were activated by an overlapping subset of allergen extracts (*A. fumigatus*, cockroach, HDM, *A. oryzae* and *B. Licheniformis*), we focused on these cytokines and allergens in more detail. As **Figures 5A**, **6A** illustrate, IL-1α and IL-36β were activated over a wide concentration range of the majority of the latter allergen extracts, although it should be noted that whereas *B. Licheniformis* failed to enhance IL-1α activity it robustly activated IL-36β (**Figures 4B**, **6A**) and the reverse was true for *A. oryzae* (**Figures 3B**, **5A**). Proteolysis of IL-1α and IL-36β in the presence of allergen extracts was also readily detected (**Figures 5B, C**, **6B**). Of note, the patterns of proteolysis of IL-36β observed with the four different allergen extracts were subtly different (**Figure 6B**), suggesting that the proteases associated with these allergens process IL-36β at distinct sites within the N-terminal pro-domain region of this cytokine.

### Mapping of Allergen-Associated Protease Cleavage Sites in IL-36β

To map some of the allergen processing sites within hIL-36β, we incubated full-length hIL-36β with Cockroach, House dust mite, *A. fumigatus*, *A. oryzae* or *B. licheniformis* extracts (**Figure 6B**) and subjected the cleavage products to N-terminal sequencing by Edman degradation. This revealed that HDM and cockroach extracts generated a new IL-36β N-terminus starting at Glu6, suggesting that proteases associated with these allergens cleaved after Arg5 (**Figures 6B, C**). Of note, we have previously identified Arg5 as the cleavage site employed by Cat G to activate hIL-36β (17). In contrast, *A. oryzae* extracts generated a new N-terminus at Arg5, suggesting a cleavage site after Gln4 (**Figure 6C**). N-terminal sequencing of cleavage products generated by *B. licheniformis* and *A. fumigatus* generated more cryptic sequencing data, suggesting multiple alternative sites within a similar region of the hIL-36β prodomain.

**Figure 7:**
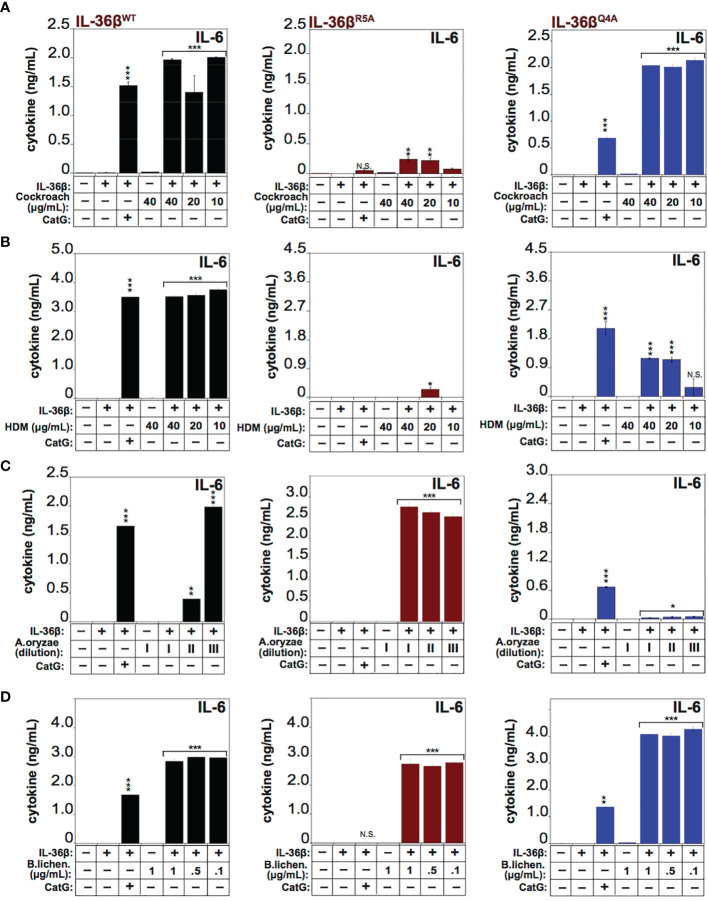
IL-36β point mutants resist activation by specific allergens HeLa^IL-36R^ cells were stimulated with 2 nM of wild type recombinant full length human IL-36β, IL-36β^R5A^, or IL-36β^Q4A^, either untreated, or pre-incubated for 2 h at 37°C with a titration of 40, 20 and 10 μg/ml of extract from Cockroach **(A)** HDM **(B),** 1:2000, 1:10000 and 1:20000 dilutions from a stock of purified protease from *A. oryzae* **(C)** or 1, 0.5 and 0.01 μg/ml of *B. licheniformis* extract **(D)**. As a control, either wild type recombinant full-length human IL-36β, IL-36β^R5A^ and IL-36β^Q4A^ cytokines were activated with 50 nM of cathepsin G. After 24h, IL-6 cytokine concentrations in cell culture supernatants were measured by ELISA. ***p < 0.001, **p < 0.01, *p < 0.1, Student’s t test. N.S., Not Significant.

To explore the requirement for proteolysis at some of these sites for activation of IL-36β by the latter allergens, we generated point mutations at Q4 (Gln4) and R5 (Arg5) within IL-36β and both mutants were soluble and expressed similar to wild type IL-36β (**Supplementary Figure 1**). We next explored the processing and activation of wild-type IL-36β, versus the Q4 and R5 IL-36β point mutants, by four of the allergens. As **Figures 7A, B** illustrates, mutation at Arg5 almost completely suppressed activation of IL-36β by Cockroach and HDM extracts, whereas this point mutant was still robustly activated by extracts from *A. Oryzae* and *B. licheniformis* (**Figures 7C, D**). In sharp contrast, mutation at Q4 had only a modest inhibitory effect on activation of IL-36β by HDM extracts, and even enhanced processing by Cockroach extract, but completely suppressed activation by *Aspergillus Oryzae* extract*s* (**Figures 7A–C**). However, both mutants were readily activated by extracts from *B. licheniformis* (**Figure 7D**), suggesting either that the protease(s) associated with the latter allergen activated IL-36β through processing at an alternative site, or alternatively that introduction of individual point mutations are insufficient to afford resistance towards proteolysis by *B. licheniformis*-associated proteases. It is also possible that there is redundancy at the Q4 and R5 sites with respect to proteolysis by proteases associated with the latter allergen. All of the preceding observations were confirmed using IL-8 production as an alternative readout of IL-36 cytokine activity (**Supplementary Figure 2**).

## Discussion

Here we have shown that diverse allergen-associated proteases can robustly process and activate human IL-1α as well as all three human IL-36 family cytokines *in vitro*, with hIL-36β and hIL-36γ being markedly more susceptible to processing and activation by the latter allergens. Taken together with previous studies which have demonstrated that IL-33 is also highly susceptible to processing and activation by allergen-associated proteases (9, 10) and a recent report which demonstrates that human and murine IL-36γ can be processed by *Aspergillus fumigatus* protease (29), this suggests that IL-1 family cytokines may have evolved to function as sentinels for aberrant protease activity that is typically associated with infection or tissue damage (reviewed in ref. 11). However, this protease-sensing function can also be adventitiously triggered *via* contact with proteases associated with harmless entities such as allergens.

Allergic responses are predominantly associated with type 2 immunity, typified by production of IL-4, IL-5 and IL-13. However, IL-36 family cytokines are capable of driving type 3 immune responses, associated with the production of IL-17 and the activation of antimicrobial effector responses (3). In light of the differences between type 2 and type 3 immunity, the processing and activation of IL-36 cytokines by multiple allergens is somewhat surprising. However, as discussed at length below, the N-termini of IL-1 family cytokines are relatively promiscuous with respect to the proteases that are capable of processing these inhibitory regions to liberate the activity of these cytokines. Thus, the likelyhood of individual IL-1 family members becoming processed and activated by allergen-associated proteases may simply be related to the most common route of entry of these allergens, which is the lung and gastrointestinal tract for the majority of allergens. Thus, the type of immune response initiated by a specific allergen may simply be a function of the most abundant IL-1 family cytokine encountered in the tissue exposed to the allergen. Because the lung is highly enriched in IL-33, which is an efficient driver of IL-5 production, eosinophil recruitment and IL-4 production downstream, the type 2 inflammation associated with many allergens may be due to the enrichment of a Th2-polarizing IL-1 family member (IL-33) at the most common route of entry. However, should allergens, or helminth worms, enter *via* the skin or another tissue enriched in IL-36 family cytokines, it may well be that a type 3 immune response may be the dominant response seen in this context.

IL-1 family cytokines all contain N-terminal pro-domains of variable length that serve as inhibitory regions to suppress the activity of these cytokines. As many prior studies have demonstrated (reviewed in (3, 14)), all IL-1 family members can be processed and activated by diverse proteases that are not typically present (at least in their active forms) in the intracellular or extracellular space. Three major sources of protease activity have emerged as effectors of IL-1 family cytokine processing (reviewed in Ref. 11). *Intracellular proteases*, such as caspases and calpains, which are activated as a consequence of injury-initiated necrosis, or pathogen-driven inflammasome activation, that typically results in programmed necrosis (pyroptosis). *Extracellular proteases*, such as elastase, cathepsin G, mast cell chymase, cathepsin S or thrombin, that are secreted by first responder cells of the immune system (e.g. neutrophils, macrophages, mast cells or keratinocytes) in response to tissue damage or pathogen detection. And *non-self proteases*, such as the *Streptococcal* protease SpeB, the *Pseudomonas* protease LasB, or the house dust mite protease Der p1, that are associated with infectious agents or allergens (29, 32, 33).

It is interesting to contemplate why all cytokines within the IL-1 family require proteolytic processing for activation. One possibility is that, given the powerful pro-inflammatory effects that IL-1 family cytokines are capable of exerting, the requirement for a proteolytic activation step represents an additional level of control that is imposed upon these cytokines to ensure that they are only activated under appropriate circumstances. However, given the striking diversity of proteases (which includes intracellular, extracellular, microbial and allergen-derived sources) that can process and activate IL-1 family members, an alternative possibility is that this cytokine family has evolved specifically to serve as sentinels for aberrant protease activity that is frequently associated with infectious agents or liberated by barrier tissues or cells of the innate immune system in response to infection or tissue damage (11). Thus, to complement the suite of ‘pattern recognition receptors’ (PRRs) that are trained upon ‘non-self’ molecular structures that indicate the presence of pathogens, IL-1 family cytokine receptors may serve as ‘activity recognition receptors’ (ARRs) for enzymatic activity (in this instance protease activity) that serves as a proxy for the detection of infection or tissue necrosis (reviewed in Ref. 11).

Although pattern recognition receptors play a critical role in sensing conserved patterns shared by many infectious agents and translating this into robust immune responses, certain pathogens, such as helminth worms, do not appear to have strong PAMPs that readily permit their detection. It has been suggested that protease activity may serve as a proxy for the detection of helminth worms by the immune system (4–6), and that the protease activity associated with common allergens may be the reason that such agents trigger paradoxically strong inflammatory responses despite their harmless nature. Thus, in addition to the detection of conserved ‘non-self’ molecular *patterns*, the immune system may have co-evolved a system to detect aberrant molecular *activities* that have the potential to perturb normal tissue and cellular homeostasis, as a proxy for the detection of pathogen activity or tissue necrosis (11). This concept is well established in plants, where numerous NLR proteins are involved in sensing diverse enzymatic activities (including proteolytic activity) associated with pathogens, in a process that has been termed ‘effector-triggered immunity’ (reviewed in (34, 35)). Similarly, the mammalian NLR protein, NLRP1, is also capable of directly sensing and responding to pathogen-associated proteases (36).

Thus, IL-1 family cytokines may have evolved as sensors for protease activity associated with infectious agents or tissue injury that couple the detection of abnormal protease activity to the instigation of inflammatory responses that coordinate wound healing as well as pathogen clearance. The emergence of multiple members of this family, as a consequence of gene duplication and divergence, would have been advantageous by permitting expansion of the range of protease activities that could be detected through the increased diversity of N-terminal pro-domains available to be tuned towards specific tissue disturbances.

In summary, here we have shown that human IL-1α and IL-36β, as well as human IL-36α and IL-36γ to a lesser degree, were highly susceptible to processing and activation by proteases associated with diverse fungal, bacterial, plant and insect allergens *in vitro*. Thus, IL-33, IL-1α and IL-36 cytokines may serve as sensors of protease activity associated with common environmental allergens, suggesting that responsiveness to exogenous proteases is a conserved property of multiple IL-1 family cytokines.

## Experimental Procedures

### Reagents

Anti-IL-1α (AHP281G) was obtained from AbD Serotec (UK). Purified neutrophil-derived cathepsin G was purchased from Calbiochem (UK). Purified neutrophil-derived elastase was purchased from Serva (Germany). Extracts of house dust mite (*Dermatophagoides pteronyssinus*), oriental cockroach (*Blatta orientalis*), pollen (Ragweed, *Ambrosia artemisiifolia*; Timothy, *Phleum pratense*, Rye, *Secale cereale*), and culture filtrates of fungi (*Alternaria alternata*; *Aspergillus fumigatus*) were obtained from Greer Laboratories. Protease from Aspergillus oryzae (EC 232-752-2) and subtilisin A protease from *Bacillus licheniformis* (EC 3.4.21.62) were purchased from Sigma (Ireland) Ltd.

### Cell Culture

HeLa cells were cultured in RPMI media (Gibco), supplemented with 5% fetal calf serum (FCS). HeLa.IL-36R cells were generated by transfection with pCXN2.empty or pCXN2.IL-1Rrp2 (IL-36R) plasmids followed by selection using G-418 antibiotic (Sigma), as previously described (17).

### Expression and Purification of Recombinant IL-1α and IL-36 Cytokines

Full-length IL-1α, IL-36α, IL-36β and IL-36γ proteins were generated by cloning the human coding sequences in-frame with the poly-histidine tag sequence in the bacterial expression vector pET45b, as previously described (17, 29). Proteins were expressed by addition of 600 μM isopropyl β-D-1-thiogalactopyranoside (IPTG) to exponentially growing cultures of *E.coli* (BL21 strain), followed by incubation for 3 h at 37 °C. Bacteria were lysed by sonication and proteins were captured using nickel-nitrilotriacetic acid (NTA) agarose (Amintra), followed by elution into PBS (pH 7.2) containing 100 mM imidazole. Eluted proteins were analyzed by SDS-PAGE electrophoresis and visualized by Coomassie blue staining.

### Cytokine Activation Assays

Reactions (40-100 μl final volume) were carried out in protease reaction buffer (50 mM Hepes, pH 7.4, 75 mM NaCl, 0.1% CHAPS) for 2 h at 37°. For IL-36 bioassays, IL-36 cytokines were typically cleaved at a 50 nM concentration and subsequently diluted onto target cells at a final concentration ranging from 0.25 nM to 2 nM.

### Site-Directed Mutagenesis

Site-directed mutagenesis was carried out using the QuikChange kit (Stratagene). Mutagenesis of IL-36 genes was verified by sequencing (Eurofins MWG Operon).

### Coupled *In Vitro* Transcription/Translation Reactions

*In vitro* transcription/translation reactions were carried out using 1 μg of purified plasmid template added to a rabbit reticulocyte lysate system (Promega, UK) for 1 h at 30 °C. Briefly, 1 μg of pET45.IL-1α expression plasmid was incubated for 1 h at 30°C in a total volume of 50 μl containing T7 polymerase, 1 μI of translation grade ^35S^methionine (1000, μCi/ml; Amersham), 50% rabbit reticulocyte lysate, RNAsin and an amino acid mixture lacking methionine. Reaction products were then aliquoted and stored at -70°C until required. ^35S^-labeled IL-1α was included in proteolysis reactions as indicated in the accompanying figure legend and reactions were then analysed by SDS-PAGE, followed by fluorography.

### Western Immunoblotting

Recombinant IL-1α, and IL-36 proteins were analysed by addition of SDS-PAGE loading buffer (2% SDS, 50 mM Tris-HCl, pH 6.8, 10% glycerol, 2.5% β-mercaptoethanol), boiled for 7 minutes and electrophoresed on 12% SDS-PAGE gels. Proteins were transferred onto 0.2 μM nitrocellulose membrane at 40 mA overnight. Membranes were blocked for 1 h (5% NFDM, 0.05% sodium azide in Tris-buffered saline, Tween-20, TBST). Membranes were probed with specific antibodies, diluted 1:1000. Membranes were washed 3 times in TBST and then incubated with anti-mouse HRP-conjugated secondary antibody (Jackson Labs, USA) diluted 1:1000. Membranes were again washed and proteins were visualized with SuperSignal West Pico (Thermo Scientific) and exposure to autoradiography film.

### Measurement of Cytokines and Chemokines

Cytokines and chemokines were measured from cell culture supernatants using specific ELISA kits (human IL-6, IL-8 obtained from R&D systems (UK). All cytokine assays were carried out using triplicate samples from each culture.

### N-Terminal Sequencing

For N-terminal sequencing, cytokine cleavage products were ran out on 15% SDS-PAGE gels, followed by immunoblotting onto PVDF membranes. Membranes were then stained with 0.02% Coomassie Brilliant blue in 40% methanol, 5% acetic acid for 20-30 seconds, followed by destining in 40% methanol, 5% acetic acid for 1 min. Membranes were rinsed in three times for 5 min in distilled water, dried between Whatman papers, followed by excision of cleavage products which were then subjected to N-terminal sequencing (Cambridge Peptides, UK).

### Quantification and Statistical Analysis

Error bars are represented as mean ± SEM of triplicate measurements from representative experiments. Statistical significance was calculated by Student’s t-test. Statistical tests were performed in Microsoft Excel using the TTEST function. Bar graphs were plotted as mean ± SEM and statistical significance was denoted as follows: *** = P < 0.001, ** = p < 0.01, * = P < 0.1.

## Data Availability Statement

The original contributions presented in the study are included in the article/**Supplementary Material**. Further inquiries can be directed to the corresponding author.

## Author Contributions

VF: Designed and carried out experiments and prepared figures. ZN: Carried out experiments. PD: Prepared figures. GS: Carried out preliminary experiments. SM: Obtained funding, designed experiments, planned the study, wrote the manuscript. All authors have contributed to the article and approved the submitted version.

## Funding

The Martin laboratory is supported by grants from the Irish Research Council Advanced Laureate programme (IRCLA/2019/133), The European Research Council Advanced Grant Programme (101020534, DESTRESS) and Science Foundation Ireland (14/IA/2622).

## Conflict of Interest

The authors declare that the research was conducted in the absence of any commercial or financial relationships that could be construed as a potential conflict of interest.

## Publisher’s Note

All claims expressed in this article are solely those of the authors and do not necessarily represent those of their affiliated organizations, or those of the publisher, the editors and the reviewers. Any product that may be evaluated in this article, or claim that may be made by its manufacturer, is not guaranteed or endorsed by the publisher.

## Supplementary Material

Supplementary Figure 1 Purification of wild-type IL-36β and IL-36β point mutants. **(A)** SDS-PAGE analysis of human recombinant full-length wild type IL-36β, IL-36β^R5A^ and IL-36β^Q4A^ mutants.

Supplementary Figure 2 IL-36β point mutants resist activation by specific allergens. HeLa^IL-36R^ cells were stimulated with 2 nM of wild type human recombinant full length IL-36β, IL-36β^R5A^, or IL-36β^Q4A^, either untreated, or pre-incubated for 2 h at 37°C with a titration of 40, 20 and 10 μg/ml of extract from Cockroach **(A)** HDM **(B),** 1:2000, 1:10000 and 1:20000 dilutions from a stock of purified protease from *A. oryzae* **(D)** or 1, 0.5 and 0.01 μg/ml of *B. licheniformis* extract **(D).** As a control, either wild type recombinant full length IL-36β, IL-36β^R5A^ and IL-36β^Q4A^ cytokines were activated with 50 nM of cathepsin G. After 24h, IL-8 cytokine concentrations in cell culture supernatants were measured by ELISA. Error bars represent the mean of triplicate determinations from representative experiments. ***p < 0.0001, **p < 0.001, *p < 0.1, Student’s t test.
