# Supplementary figures and images for: IL-1α and IL-36 Family Cytokines Can Undergo Processing and Activation by Diverse Allergen-Associated Proteases
Source: Front Immunol. 2022 Jun 30;13:879029. doi: 10.3389/fimmu.2022.879029 (PMC9280268; doi:10.3389/fimmu.2022.879029)

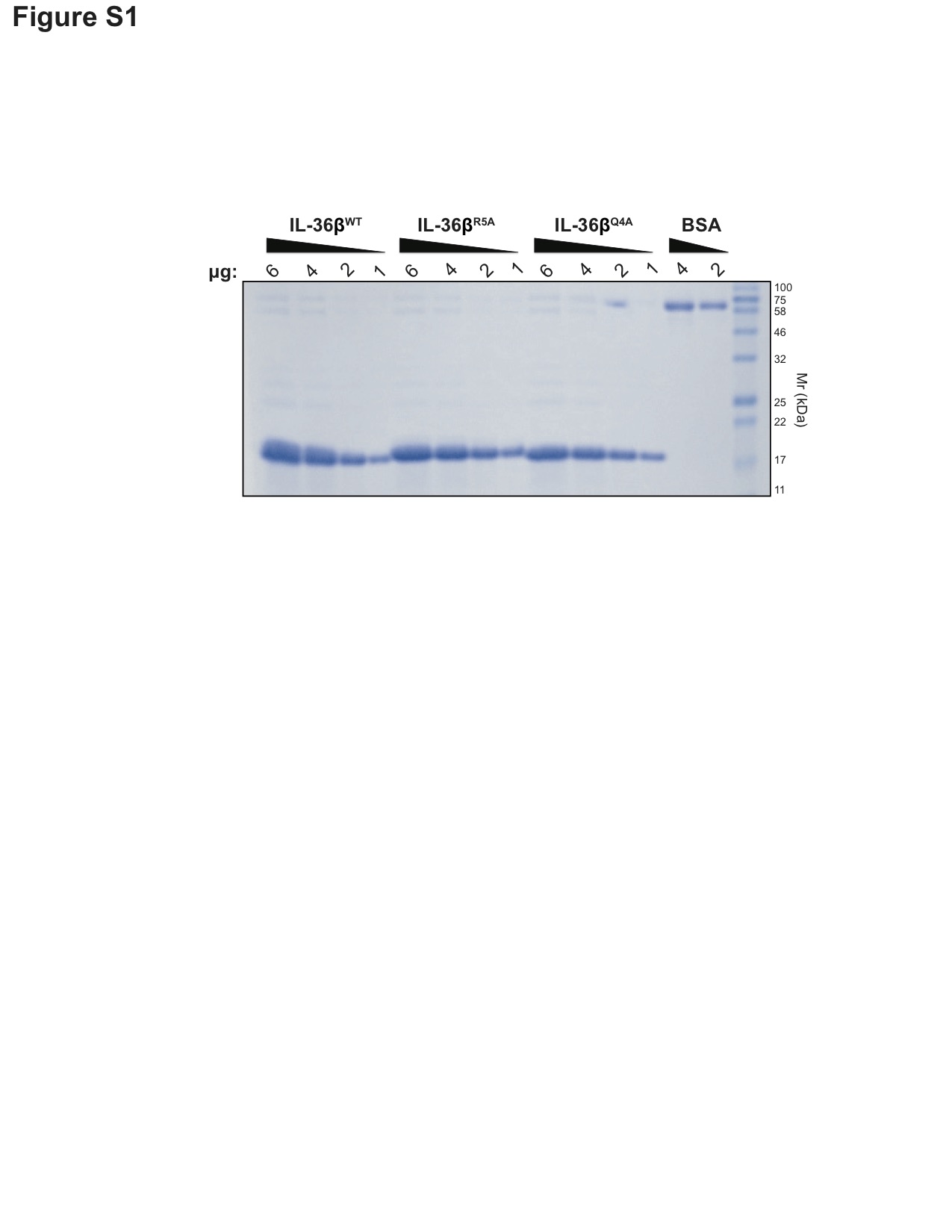

Supplement: Supplementary Figure 1 — Purification of wild-type IL-36β and IL-36β point mutants. (A) SDS-PAGE analysis of human recombinant full-length wild type IL-36β, IL-36βR5A and IL-36βQ4A mutants. [file Image_1.jpeg]

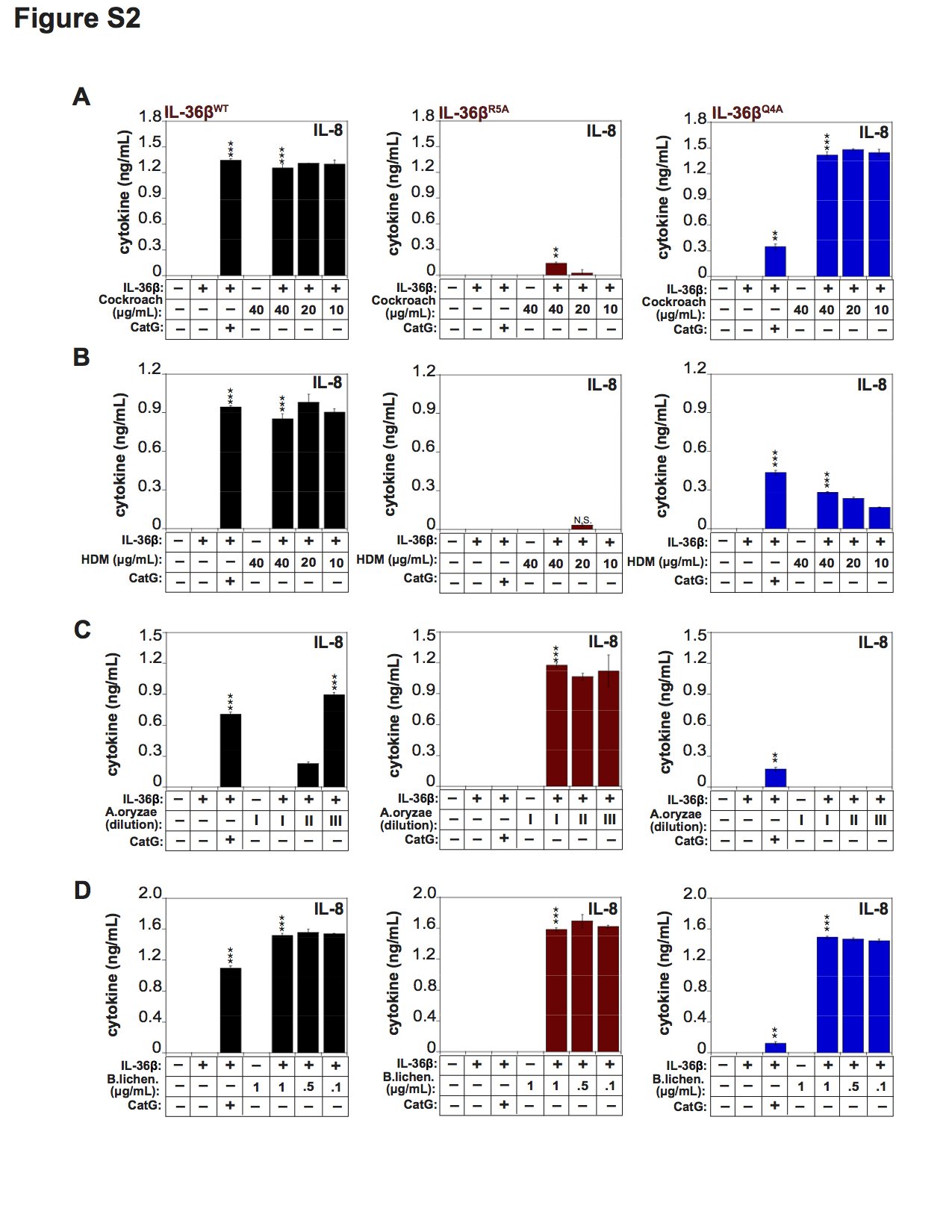

Supplement: Supplementary Figure 2 — IL-36β point mutants resist activation by specific allergens. HeLaIL-36R cells were stimulated with 2 nM of wild type human recombinant full length IL-36β, IL-36βR5A, or IL-36βQ4A, either untreated, or pre-incubated for 2 h at 37°C with a titration of 40, 20 and 10 μg/ml of extract from Cockroach (A) HDM (B), 1:2000, 1:10000 and 1:20000 dilutions from a stock of purified protease from A. oryzae (D) or 1, 0.5 and 0.01 μg/ml of B. licheniformis extract (D). As a control, either wild type recombinant full length IL-36β, IL-36βR5A and IL-36βQ4A cytokines were activated with 50 nM of cathepsin G. After 24h, IL-8 cytokine concentrations in cell culture supernatants were measured by ELISA. Error bars represent the mean of triplicate determinations from representative experiments. ***p < 0.0001, **p < 0.001, *p < 0.1, Student’s t test. [file Image_2.jpeg]
